# The effect of breastfeeding education with grandmothers’ attendance on breastfeeding self-efficacy and infant feeding pattern in Iranian primiparous women: a quasi-experimental pilot study

**DOI:** 10.1186/s13006-020-00325-5

**Published:** 2020-10-12

**Authors:** Tayebeh Gharaei, Leila Amiri-Farahani, Shima Haghani, Syedeh Batool Hasanpoor-Azghady

**Affiliations:** 1 Department of Reproductive Health and Midwifery, Nursing Care Research Center, School of Nursing and Midwifery, Iran University of Medical Sciences, Tehran, Iran; 2 Department of Biostatistics, Nursing Care Research Center, Iran University of Medical Sciences, Tehran, Iran

## Abstract

**Background:**

One of the most important factors that affects breastfeeding self-efficacy and exclusivity is breastfeeding support provided by the family. The aim of this study was to determine the effect of breastfeeding education sessions for primiparous women, with and without the attendance of maternal grandmothers, on breastfeeding self-efficacy and infant feeding patterns.

**Methods:**

This quasi-experimental study was conducted on 64 primiparous women who referred to the Antenatal Clinic of Amiralmomenin Hospital, Tehran, Iran from June to December, 2018. Eligible pregnant women were allocated into two groups; either with and without grandmothers in attendance. Group assignments were determined according to the week the women had prenatal care at the hospital. All eligible women seen in the clinic during 1 week were assigned to one group and women who presented in the alternating week were assigned to the other group. In the education group with grandmothers in attendance, each woman participated in two prenatal education programs with her mother and one postpartum program approximately 3 h before discharge. In the other group, participating mothers attended breastfeeding education sessions without the grandmother’s attendance. The participating mothers answered questions from the Breastfeeding Self-Efficacy Scale-Short Form (BSES-SF) at the time of the hospital discharge, and 4 and 8 weeks after delivery. Questions about the infants’ feeding patterns were asked by telephone contact with the participating mothers from both groups at the same time periods.

**Results:**

The mean BFSE scores were signficantly different between the groups with grandmothers and without grandmothers at the time of the hospital discharge (60.15 ± 4.47 vs. 56.84 ± 6.22, independent t-test; *p* = 0.017), and at 4 weeks (61.71 ± 2.66 vs. 56.62 ± 9.12, *p* = 0.004) and 8 weeks after delivery (63.68 ± 2.14 vs. 60.03 ± 6.32, *p* = 0.003). No significant difference existed in infant feeding patterns between the groups at the same time periods.

**Conclusions:**

This study suggests that breastfeeding education with grandmothers’ attendance is effective in improving the mothers’ breastfeeding self-efficacy. A family-centered program should be considered in beastfeeding education for increasing of exclusive breastfeeding.

## Background

The World Health Organization (WHO) recommends exclusive breastfeeding (EBF) for 6 months and its continuation up to 2 years of age [1]. One of the targets of the global strategy for infant and young child feeding is EBF for at least 50% of babies under 6 months of age until 2025 [2].

According to the WHO and United Nations International Children’s Emergency Fund (UNICEF), the global rates of EBF are not ideal and about 40% of six-month-old infants are exclusively breastfed [3]. In Iran, the EBF rate for six-month-old infants was 44% in 2000 [4], which decreased to 27.7% in 2006 and it was measured since birth [5]. Based on a systematic review and meta-analysis study, the overall prevalence of EBF was 53% in 2018. In Iran, health policy and decision makers try to take interventions that encourage mothers to breastfeed their infants exclusively [6].

Studies show breastfeeding self-efficacy (BFSE), family support, mothers’ education, age, economic status of the family, and literacy are among the most prominent factors that affect EBF [7], with BFSE reflecting maternal self-confidence and ability to breastfeed [8]. Mothers with high levels of self-efficacy were more determined to breastfeed and they responded appropriately to breastfeeding problems [9]. The results of studies show that there is a direct correlation between mother’s BFSE and rate of exclusive breastfeeding. According to these studies, mothers with higher self-efficacy scores exclusivelybreastfed infants compared with those who had lower self-efficacy scores [7, 10, 11]. BFSE is thought to be affected by four main factors: observation of other women breastfeeding, mother’s perception of support for breastfeeding, previous breastfeeding experience, and physiological responses such as fatigue, fear, and anxiety [12]. Studies suggest that one of the most important factors that affects the onset of lactation and BFSE is breastfeeding support by the family during the early postpartum period [13, 14]. Heidari et al. have reported that if the family members have appropriate knowledge and experience in breastfeeding, they will play an efficient supportive role in the mother’s attempts to breastfeed [15].

Grandmothers provide key support and encouragement for mothers to breastfeed because their knowledge, experience, and attitude affect the mothers’ decision to begin and continue breastfeeding [16, 17]. In Australia, a small randomized controlled trial was conducted with two groups, the intervention (mothers and grandmothers) and control (mothers without grandmothers). According to the results, the intervention increased the duration of EBF by 67 days for the group which included grandmothers and 46 days for the group which did not include grandmothers [18]. However, the results of another study showed that grandmother’s support of the new mother may cause her to discontinue breastfeeding [19]. In Brazil, the results of three studies indicated that grandmother’s support, especially maternal grandmothers, might reduce the duration of exclusive breastfeeding [20–22]. In Kuwait, grandmothers often encourage mothers to formula feed their infants. The grandmothers are reassured that the baby is satisfied and formula feeding stops their hunger cries [23]. Grandmothers’ attitudes towards breastfeeding are influenced by their lactation experiences [24]. The women whose mothers had breastfed or had a positive attitude toward breastfeeding, were more likely to breastfeed their infants exclusively and to continue breastfeeding [25]. Studies show grandmothers play an important role in the decision-making process for infant feeding; hence, they should be involved in education sessions for breastfeeding promotion [18, 26]. In terms of the importance of EBF promotion, Iranian maternal grandmothers play a key role during the early postnatal period. They provide care for their daughters after the delivery, in addition to emotional and informational support. Therefore, the mother can use the grandmother’s recommendations about feeding her baby. Grandmother’s participation seems be necessary in educational sessions [15, 27].

The most important necessities for performing the current research included proposals from previous researches where inclusion of grandmothers in antenatal breastfeeding education might help address the biases/judgements towards breastfeeding and give grandmothers the necessary knowledge/skills to offer effective breastfeeding support [18–23]. In Iranian culture, a maternal grandmother provides care for her daughter during the first 10 days after delivery. Their partners frequently have difficulty taking time from work and often do not attend antenatal education sessions. Therefore, the purpose of this research was to determine the effect of breastfeeding education sessions for primiparous women, with and without the presence of maternal grandmothers, on breastfeeding self-efficacy and infant feeding patterns.

## Methods

### Design and participants

This was a two-group quasi-experimental clinical trial study. Participants included primiparous women and their mothers who attended the prenatal care clinic of Amiralmomenin Hospital, Islamic Azad University of Tehran, Iran. The recruitment of pregnant women took place for 4 weeks in June 2018.

The intervention began in July 2018. Each participant was followed until 8 weeks after delivery. The follow-up period finished in December 2018. The sampling process for eligible pregnant women was carried out on a weekly, continual basis. Inclusion criteria consisted of the ability to understand and speak Persian by the participating mothers and grandmothers, age range of the participating mothers from 18 to 35 years, having a mother (maternal grandmother), primiparity, low-risk pregnancy, 31–34 weeks of gestational age, and delivery of the infant at Amiralmomenin Hospital. Exclusion criteria were contraindications to breastfeeding that included use of chemotherapy drugs, ergotamines, herpes lesions on the breast, AIDS, and severe depression during pregnancy. Withdrawal criteria consisted of lost to follow-up, delivery earlier than 37 weeks of gestational age (prematurity), hospitalization in the neonatal intensive care unit (NICU), the absence of mother or grandmother for more than one education session, and the unavailability of the maternal grandmother during the mother’s hospital stay and for 10 days after delivery.

The pregnant women who presented with their mothers to the clinic were approached for study enrollment. Those who met the inclusion criteria and expressed interest in participating signed the written informed consent form. The researcher allocated participants to either the education group with the grandmothers in attendance or an education group without the grandmothers in attendance. Assignment to the groups was determined by the week in which the participating mothers had prenatal care at the hospital. All eligible women seen in the clinic during the one-week period were assigned to the group with grandmother’s attendance and eligible participating mothers who presented to the clinic in the alternating week were assigned to the group without the grandmother’s attendance. In the education group with grandmother’s attendance, each woman and her mother (maternal grandmother) participated in groups of four (two participating mothers and two grandmothers). Each woman participated in two prenatal education programs with her mother (maternal grandmother) and one postpartum program approximately 3 h before discharge. In the second group (without grandmothers), the participating mothers also participated in small groups of four mothers per group. Breastfeeding education sessions were held in a breastfeeding education clinic at Amiralmomenin Hospital. Each session lasted for 1 h.

Trial registration number: IRCT20180427039436N1. Registered 12 June 2018.

### Intervention

The first and second parts of the breastfeeding education sessions were provided by the researcher when the participating mothers were at 31–34 and 35–37 weeks gestation, respectively. The contents of breastfeeding education were designed according to a guide book for monitoring baby-friendly hospitals in Iran [28–30].

The information provided in educational sessions were the same for both groups. The educational program for both groups at 31–34 weeks gestation consisted of the benefits of human milk, the importance of EBF, symptoms of starvation and fullness, breastfeeding on demand, breastfeeding from either breast, showing different positions of breastfeeding using a doll, the latch-on, and finally erroneous beliefs on breastfeeding and their related corrections. Erroneous beliefs that were discussed with the participants in both groups and grandmothers in the grandmother group included: colostrum due to jaundice in the baby, use of a pacifier to calm the baby, superiority of formula compared to human milk, cessation of breastfeeding due to breast nipple soreness and mastitis, limitations on duration of breastfeeding per meal, infant′s restlessness and awakenings at night due to inadequacy of human milk, and the watery milk of some mothers.

The breastfeeding education at 35–37 weeks of gestation included breast pumping, common breast problems and their prevention and treatment, ways to diagnose the adequacy of milk, support for the lactating mother, ways to increase the amount of milk, complications with the use of a bottle, formula, and pacifier, diseases and breastfeeding, and drug use during breastfeeding. The other points about erroneous beliefs on breastfeeding included: milk accumulation in breasts due to delayed breastfeeding, impossibility of breastfeeding continuity due to employment by the mother and baby’s illness, giving water to infants less than 6 months of age, and the lack of milk storage [28, 30].

At the end of each session, the participants’ questions were answered. The participants received colorful, easy to understand pamphlets that had been approved by the Education Committee at Amiralmomenin Hospital.

The postnatal session included a review of prenatal education programs, presentation of healthy baby software provided by the Ministry of Health and Medical Education (MOHME), emphasis of practical education, and assistance for mothers to breastfeed their infants. The postnatal session was held for the participating mothers and maternal grandmothers in the grandmother group and only for the participating mothers in the other group, approximately 3 h before discharge. The groups were located in separate rooms of the maternity ward. In both groups, the maternal grandmothers were present in the hospital to assist with care of their daughters after childbirth. After discharge (during 10 days after delivery), the researcher contacted the participating mothers in both groups by telephone to determine if the grandmothers continued this care. In any case, the participants could contact the researcher if they had breastfeeding issues.

### Sample size

An adequate sample size was difficult to determine because of the lack of preliminary studies. Therefore, we used a pilot study design to test the feasibility of the intervention, instead of attempting to determine a difference when we did not recruit the appropriate number of samples according to the MRC Complex Intervention Framework [31]. In the pilot study, the sample size was determined to be 70 individuals according to α = 0.05, β = 0.2, effect size (ES) = 0.7, and 10% possibility of dropouts (*n* = 35 samples in each of the control and experiment groups).

$$ n=\frac{2{\left({z}_1+{z}_2\right)}^2}{ES^2}=\frac{2{\left(1.96+0.84\right)}^2}{(0.7)^2}=32 $$

### Data collection

We used participant characteristics form, the breastfeeding self-efficacy scale-short form (BSES-SF), and infant feeding patterns to collect data.

### Participant characteristics form

The first part of the participant characteristics form included the age, education, occupation, and economic status of the participating mothers. In both groups, the maternal grandmothers could also complete the information themselves if they were present with their daughters for prenatal care in the hospital and met the inclusion criteria. The grandmothers’ information consisted of their age, education level, occupation, number of breastfed children, parity number, and breastfeeding experiences.

The second part included information about obstetric history (gestational age in recruitment, gestational age at birth, and mode of delivery (normal vaginal delivery, cesarean section or vacuum extraction). The aforementioned parts of the participant characteristics form were completed at 31–34 weeks gestation. The number of breastfeeding education sessions that the participating mothers and grandmothers participated in were completed after delivery.

The third part of the participant characteristics form pertained to support for the mother from her husband in childcare activities that included changing diapers, bathing, and calming the baby. The following questions were asked: “How often did your husband help you in calming the baby when he was at home?”; “How often did your husband help you in bathing the baby when he was at home?”; and “How often did your husband help you in changing diapers the baby when he was at home?”. The participating mothers completed the questions 8 weeks after the delivery. The answers were given with respect to a six-point Likert scale that ranged from never to always.

### Breastfeeding self-efficacy scale-short form (BSES-SF)

The BSES-SF consists of 14 items [32]. In this study, we applied the Persian version, which contains 13 items. The Persian version of the BSES was assessed by Araban et al. [33]. This scale uses a five-point Likert scale that ranges from absolutely agree to absolutely disagree, where the sum of scores is from 13 to 65. Araban et al. used the face and content validities to determine the validity of the Persian version of the BSES-SF. Accordingly, 30 pregnant women were interviewed to determine the level of difficulty and ambiguity of the face validity of this questionnaire. In addition, 10 reproductive health experts verified the content validity. The findings of the content and face validity showed almost perfect results. The scale reliability with respect to internal consistency was confirmed by Cronbach’s Alpha at a value of 0.91. The questionnaire was completed at hospital discharge (24 h after delivery) and at 4 and 8 weeks after delivery. The length of the hospital stay was approximately 24–48 h.

### Infant feeding pattern

The researcher collected infant feeding information to determinate the frequency of EBF at hospital discharge. Subsequent infant feeding information was collected by telephone contact with the the participating mothers at 4 and 8 weeks after delivery [29]. The question was asked as follows: “Have you ever given any ingredients to your baby other than your milk since birth?” The reasons for cessation EBF were asked, when appropriate.

According to the above question, the infant feeding pattern included: 1. EBF (only breast milk), 2. combined feeding: breast milk with formula, other milks or liquids (e.g., water, fruit juice, or candy) or foods, and 3. alternative nutrition: formula, complementary foods, and liquids other than breastmilk.

### Ethical considerations

This research was approved by the Ethics Committee of Iran University of Medical Sciences, Tehran, Iran (ethics code: IR.IUMS.REC 1396.9511373007) and registered with the Iranian Registry of Clinical Trials (ID number: IRCT20180427039436N1). Participants were fully aware of the study procedure and signed the informed consent form for study participation.

### Data analysis

The independent sample t-test was applied to compare the mean BSES-SF scores between groups and for analysis of the quantitative data. We used analysis of variance with the repeated measures for pairwise comparison. The chi-squared and Fisher’s exact tests were used for categorical variables and comparison of the infant feeding patterns in groups, respectively. The data were analyzed using SPSS software (v. 20). *P* < 0.05 was considered statistically significant.

## Results

The researcher started the recruitment of pregnant women in June 2018 and intervention began in July 2018. The follow up was finished in December 2018. A total of 83 women were assessed for eligibility. Of these, seven women did not meet the inclusion criteria and three women refused to participate. In total, 73 pregnant women were assigned to either the group with grandmother’s attendance (*n* = 35) or the group without grandmother’s attendance (*n* = 38).

**Fig. 1:**
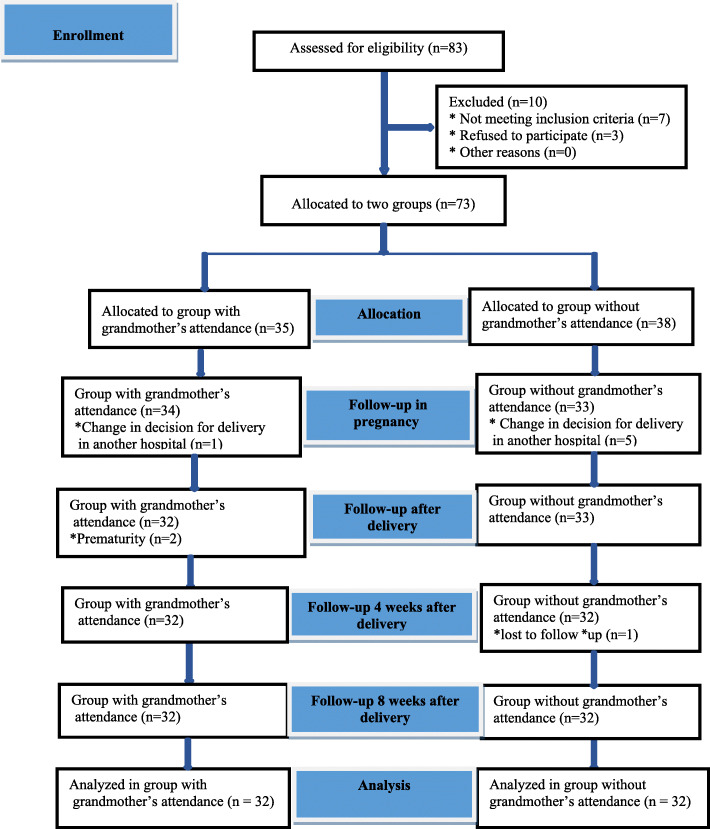
The allocation of participants into two groups, with grandmother’s attendance and without grandmother’s attendance

Based on exclusion criteria, six participating mothers were removed from the group without grandmother’s attendance due to a change in decision for delivery in another hospital (five participating mothers) and lost to follow-up due to no contact (one participant). During the study, three participating mothers were excluded from the group with grandmother’s attendance due to prematurity (two participants) and change of decision for delivery in another hospital (one participant). Finally, 64 participating mothers and grandmothers (32 in each group) were evaluated (Fig. 1).

There was no difference between the baseline characteristic of the two groups (Tables 1 and 2). All of the grandmothers from both groups had breastfeeding experience. There was a significant difference between the two groups in the numbers of children the grandmothers breastfed; however, the correlation test did not show a significant difference between this variable and BFSE at hospital discharge (*P* = 0.140), and 4 weeks (*P* = 0.381) and 8 weeks after delivery (*P* = 0.613). The independent t-test did not show a significant difference between this variable and infant feeding pattern at hospital discharge (*P* = 0.424), and 4 weeks (*P* = 0.439) and 8 weeks after delivery (*P* = 0.098). Hence, it was not considered a confounding variable.

**Table 1:** Descriptive statistics and between-group comparison of continuous participant characteristics

Variable	Groups		
	Without grandmother attendance (***n*** = 32)	With grandmother attendance (***n*** = 32)	Independent t-test ***P***-value
Mother’s age (year), Mean ± SD	24.93 ± 4.28	25.68 ± 5.01	0.522
Grandmother’s age (year), Mean ± SD	49.25 ± 5.67	48.93 ± 8.19	0.860
Parity of grandmother, Mean ± SD	4.40 ± 2.13	3.59 ± 1.29	0.071
The number of children breastfed by the grandmothers.	4.37 ± 2.13	3.12 ± 1.45	0.008
Gestational age in recruitment (week), Mean ± SD	33.63 ± 0.85	33.84 ± 0.86	0.32
Gestational age at birth (week), Mean ± SD	38.94 ± 0.75	38.69 ± 0.95	0.25

**Table 2:** Descriptive statistics and between-group comparison of categorical participant characteristics

Variable	Groups		Fisher Exact test ***P***-value
	Without grandmother attendance (***n*** = 32)	With grandmother attendance (***n*** = 32)	
	*n* (%)	*n* (%)	
Mother’s occupation			
Housewife	32 (100)	29 (9.4)	0.238
Employed	0 (0)	3 (90.6)	
Grandmother’s occupation			
Housewife	32 (100)	31 (96.9)	0.999
Employed	0 (0)	1 (3.1)	
Husband’s occupation			
Worker	6 (18.8)	4 (12.5)	0.091
Employed	1 (3.1)	7 (21.9)	
Self-employed	25 (78.1)	21 (65.6)	
Economic status of the family (month’s wages: millions of Rials)			
< 20	1 (3.1)	0 (0)	0.891
20–40	20 (62.6)	22 (68.8)	
40–100	10 (31.2)	10 (31.2)	
> 100	1 (3.1)	0 (0)	
Mother’s education			
Elementary	1 (3.2)	0 (0)	0.083
Secondary	2 (6.2)	3 (9.4)	
High school	24 (75)	16 (50)	
University	5 (15.6)	13 (40.6)	
Grandmother’s education			
Elementary	8 (25)	2 (6.2)	0.211
Secondary	15 (46.9)	18 (56.3)	
High school	7 (21.9)	8 (25)	
University	2 (6.2)	4 (12.5)	
Mode of delivery			
Normal vaginal	6 (18.8)	5 (15.6)	0.75
Vacuum extraction	1 (3.1)	0 (0)	
Cesarean section	25 (78.1)	27 (84.4)	

There was no significant difference between the two groups in terms of husband’s participation in childcare activities such as changing diapers, bathing, and calming the baby. The majority of the participating mothers in both groups stated that their husbands never helped them during changing diapers and bathing the baby. However, 53.1% of them in the group without grandmothers’ attendance and 68.8% of them in the group with grandmothers’ attendance stated that their husbands always or most of the time helped to calm the crying baby (Additional file 1: Table S1).

**Fig. 2:**
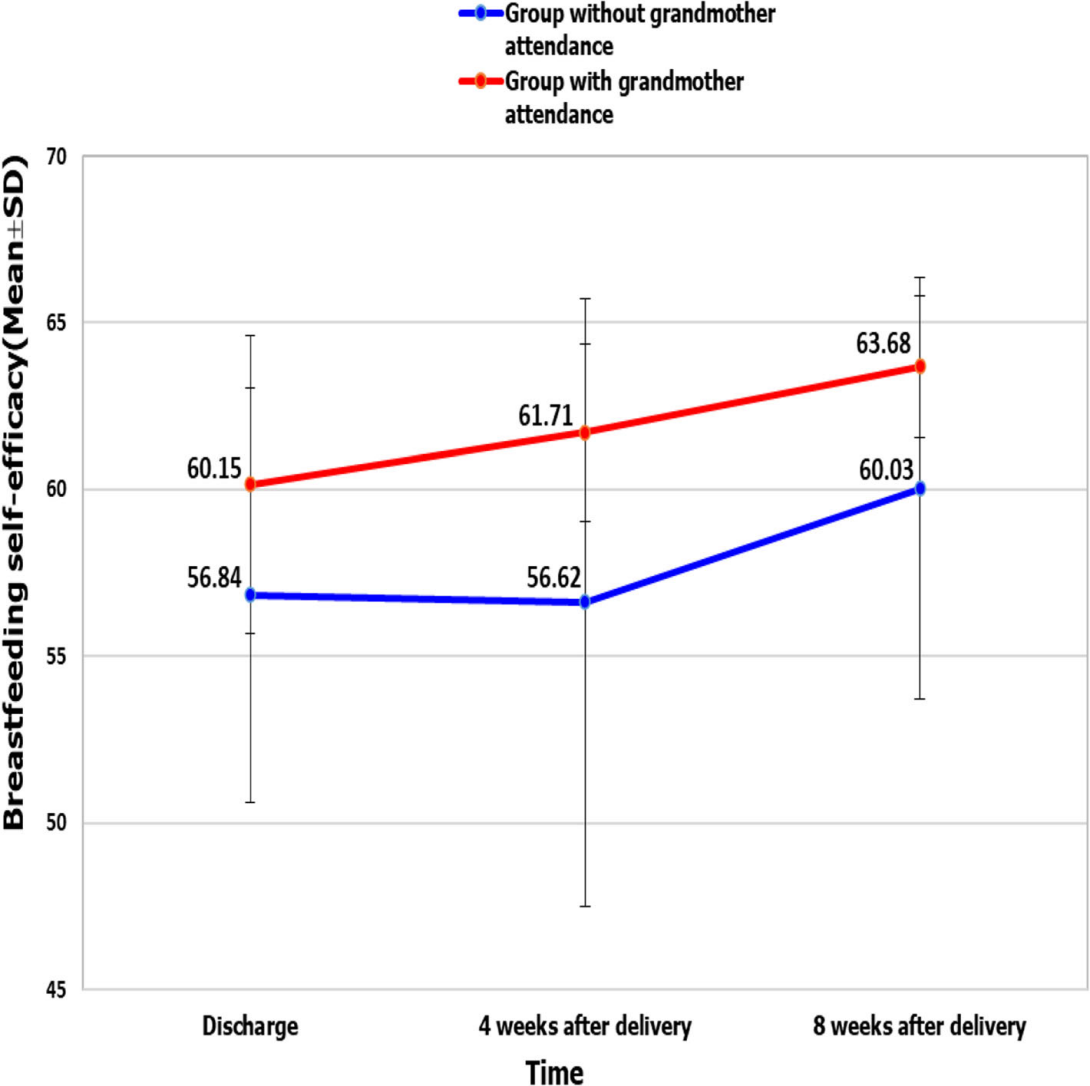
Comparison the mean of BFSE scores between the two groups at the times of hospital discharge, and four and eight weeks after delivery

The mean scores of BFSE differed between the groups with grandmothers and without grandmothers at hospital discharge (60.15 ± 4.47 vs. 56.84 ± 6.22), and 4 weeks (61.71 ± 2.66 vs. 56.62 ± 9.12) and 8 weeks after delivery (63.68 ± 2.14 vs. 60.03 ± 6.32). The independent t-test indicated that this difference was significant at hospital discharge (*p* = 0.017), and 4 weeks (*p* = 0.004) and 8 weeks after delivery (*p* = 0.003). Bonferroni’s pairwise, as a within group comparison, indicated that the mean BFSE score was significantly higher at 8 weeks (*P* < 0.001); however, there was no significant difference at the time of hospital discharge and at 4 weeks. In other words, education of the grandmothers increased the self-efficacy score of the participating mothers in the group with grandmother′s attendance to a statistically significant level, such that the BFSE increased over time (Fig. 2).

The frequency of EBF in both groups was not significantly different at hospital discharge (*p* = 0.61). The frequency of EBF was 43.8% in the group with grandmother’s attendance and 28.1% in the group without grandmother’s attendance at 4 weeks after delivery. The frequency of EBF was 25% in the group with grandmother’s attendance and 18.8% in the group without grandmother’s attendance at 8 weeks after delivery. The results of the chi-squared test demonstrated no significant difference between the two groups in terms of EBF at 4 weeks (*p* = 0.29) and 8 weeks after delivery (*p* = 0.76) (Table 3). The reasons for cessation of EBF included infant formula prescribed by pediatricians in the group with grandmother’s attendance (29%) and the group without grandmothers (40%). Other reasons included mother’s complaint due to baby’s crying (12% vs. 15%), mother’s perception of a decreased amount of milk or inadequacy of human milk (8% vs. 11%), and other family member interferences in feeding the baby such as paternal grandmother, husband, or sister (33% vs. 40%).

**Table 3:** The frequency of infant feeding patterns between the groups at hospital discharge, and four and eight weeks after delivery

Infant feeding patterns	Group without grandmother attendance (***n*** = 32)	Group with grandmother attendance (***n*** = 32)	Chi-square test ***P*** - value
	*n* (%)	*n* (%)	
Hospital discharge			
EBF^a^	29 (90.6)	31 (96.8)	0.61
Combined feeding^b^	3 (9.3)	1 (3.12)	
Four weeks after delivery			
EBF	9 (28.1)	14 (43.8)	0.29
Combined feeding	23 (71.9)	18 (56.2)	
Eight weeks after delivery			
EBF	6 (18.8)	8 (25)	0.76
Combined feeding	26 (81.2)	24 (75)	

^a^Exclusive Breastfeeding: only breast milk

^b^Combined feeding: breast milk with formula, other milks, liquids or foods

## Discussion

The purpose of this study was to determine the effect of breastfeeding education sessions for Iranian primiparous women, with and without the presence of maternal grandmothers, on mother’s BFSE and infant feeding patterns. Based on the study results, we observed significant differences in the BFSE of women whose mothers (grandmothers) attended the education classes compared to the women who attended the classes alone. While there were differences in the EBF of women in the grandmother group compared to the other group, these results were not significant.

The increase in the BFSE of women in the grandmother group is consistent with that reported by Hannula et al. They found that the inclusion of family members in the breastfeeding interventions and the mother’s encouragement to breastfeed by people close to the mother increased the mother’s BFSE at the time of discharge and thereafter [34].

In our findings, the BFSE score of the participating mothers in the group with grandmother′s attendance increased over time. The mean BFSE scores were 60.15 at the time of hospital discharge, 61.71 at 4 weeks after delivery, and 63.68 at 8 weeks after delivery. Liu et al. and Otsuka et al. reported that if a mother began to breastfeed her infant, her breastfeeding self-efficacy score would increase with time; hence, time has an effect on the mother’s BFSE [35, 36]. As a result, women get more confident and experienced, and empovered to overcome breastfeeding problems over time.

In the current study, we observed differences in the EBF of women in the grandmother group compared to the group without grandmothers at the time of the hospital discharge (96.8% vs. 90.6%), and at four (43.8% v 28.1%) and eight (25% vs. 18.8%) weeks after delivery. Although these differences were not significant, they were clinically noteworthy. The results of this study supported findings from previous studies. In Canada, Abbass-Dick et al. conducted an interventional program for mothers and fathers. Although they reported higher EBF rates for mothers in the intervention group compared with the control group, these differences were not statistically significant [37]. In another study, Abdeyazdan et al. examined the effect of breastfeeding education with maternal grandmothers’ attendance. Exclusive breastfeeding rates increased in the intervention group compared with the control group, but this finding was not statistically significant [38].

Our findings contrasted the results of previous studies that found a significant difference in exclusive breastfeeding [15, 17, 39]. In these studies, consultations and practical help that mothers received after the hospital discharge and the group discussion with family members on breastfeeding problems were the main underlying reasons for a statistically signficant increase in exclusive breastfeeding. Also, the control groups only received routine prenatal care and were not provided access to educational programs. In our study, both groups received similar educational information about breastfeeding. There is a significant correlation between the mother’s BFSE and the rate of exclusive breastfeeding [40]. So that, the results of two studies showed that mothers with high self-efficacy were significantly more to breastfeed their babies exclusively than the mothers with low self-efficacy [7, 11]. In our study, despite the increase in mother’s BFSE, the frequency of exclusive breastfeeding did not significantly increase in the group with grandmothers compared to the group without grandmothers. BFSE is one of the factors that affects exclusive breastfeeding. Other factors include attitude towards breastfeeding, initiation of breastfeeding within the first hour of delivery [41], and birthweight [42]. These factors were not investigated in our research.

Many women ceased EBF in both groups at 8 weeks postpartum. One of the reasons for ceasing EBF was that some pediatricians prescribed infant formula for the treatment of icterus. The other reasons were mother’s concern about inadequacy of her milk, other family members interferences (paternal grandmother, husband, sister) in feeding the babies and giving the infant drinking water, sugar juice, manna, and mint extract with the intent to relieve colic pains and icterus. These reasons were self-reported by the participating mothers during the telephone interviews. The aforesaid results were reported by other researchers in studies related to discontinuation of EBF by the mothers [5, 43–47].

The first limitation of our study was the lack of randomization of participants. In fact, the nonrandomized studies are more prone to systematic and confounding biases than randomized clinical trials; consequently, it is also difficult to make causal inferences about the effect of an intervention [48]. The second limitation was lack of process evaluation, which could have included an assessment of the perceived influence of the grandmothers’ support. In our study, a few mothers (equal numbers in both groups) contacted the researcher when they faced with breastfeeding problems (engorgement, mastitis, nipple sore). Hence, the third limitation was the probable effect of phone calls made by these participants on the study results. For future research, given the possibility of an effective intervention, it is recommended to perform the study with a larger sample size to assess the efficacy of the intervention. For promoting of exclusive breastfeeding, the roles of other family members should be considered.

## Conclusions

This study demonestrated that breastfeeding education with grandmothers’ attendance was an efficient approach to enhancing breastfeeding self-efficacy for primiparous women. Since the other family members affect the rate of EBF, a family-centered program should be considered in beastfeeding education for increasing of exclusive breastfeeding.

## Supplementary information

**Additional file 1: Table S1.** Husband’s support and between-group comparison of categorical participant characteristics.

## Data Availability

Not applicable.

## Abbreviations

WHO: World Health Organization
EBF: Exclusive breastfeeding
UNICEF: United Nations International Children’s Emergency Fund
BFSE: Breastfeeding self-efficacy
BSES-SF: Breastfeeding self-efficacy scale-short form
