# Supplementary material for: The effect of breastfeeding education with grandmothers’ attendance on breastfeeding self-efficacy and infant feeding pattern in Iranian primiparous women: a quasi-experimental pilot study
Source: Int Breastfeed J. 2020 Oct 12;15:84. doi: 10.1186/s13006-020-00325-5 (PMC7552372; doi:10.1186/s13006-020-00325-5)
Supplement: Supplementary file 1 — Additional file 1: Table S1. Husband’s support and between-group comparison of categorical participant characteristics. [file 13006_2020_325_MOESM1_ESM.docx]

**Additional file 1 : Table S1. Husband's support** **and between-group comparison of categorical participant characteristics**

| Fisher Exact test  P-value | Groups | | Variable |
| --- | --- | --- | --- |
|  | With grandmother attendance (n=32) | Without grandmother attendance (n=32) |  |
|  | n (%) | n (%) |  |
| 0.19 | 19 (59.4)  2 (6.2)  2 (6.2)  2 (6.2)  3 (9.4)  4 (12.5) | 16 (50)  8 (25)  4 (12.5)  0 (0)  2 (6.2)  2 (6.2) | Husbands’participation in changing diapers  Never  Seldom  Sometimes  Usually  Most of the time  Always |
| 0.89 | 24 (75)  2 (6.2)  0 (0)  1 (3.1)  1 (3.1)  4 (12.5) | 22 (68.8)  3 (9.4)  0 (0)  0 (0)  2 (6.2)  5 (15.6) | Husbands’participation in bathing the baby  Never  Seldom  Sometimes  Usually  Most of the time  Always |
| 0.33 | 1 (3.1)  3 (9.4)  4 (12.5)  2 (6.2)  10 (31.2)  12 (37.6) | 0 (0)  4 (12.5)  7 (21.9)  4 (12.5)  12 (37.5)  5 (15.6) | Husbands’participation in calming the baby  Never  Seldom  Sometimes  Usually  Most of the time  Always |
